# Manipulation of encapsulated artificial phospholipid membranes using sub-micellar lysolipid concentrations

**DOI:** 10.1038/s42004-024-01209-z

**Published:** 2024-06-01

**Authors:** Pantelitsa Dimitriou, Jin Li, William David Jamieson, Johannes Josef Schneider, Oliver Kieran Castell, David Anthony Barrow

**Affiliations:** 1 School of Engineering, Cardiff University, Queen’s Buildings, Cardiff, CF24 3AA UK; 2 School of Pharmacy and Pharmaceutical Sciences, College of Biomedical and Life Sciences, Cardiff University, Redwood Building, Kind Edward VII Avenue, Cardiff, CF10 3NB UK; 3 Institute of Applied Mathematics and Physics, School of Engineering, Zurich University of Applied Sciences, Technikumstr. 9, 8401 Winterthur, Switzerland

**Keywords:** Fluids, Biophysical chemistry, Gels and hydrogels, Fluidics, Membrane lipids

## Abstract

Droplet Interface Bilayers (DIBs) constitute a commonly used model of artificial membranes for synthetic biology research applications. However, their practical use is often limited by their requirement to be surrounded by oil. Here we demonstrate in-situ bilayer manipulation of submillimeter, hydrogel-encapsulated droplet interface bilayers (eDIBs). Monolithic, Cyclic Olefin Copolymer/Nylon 3D-printed microfluidic devices facilitated the eDIB formation through high-order emulsification. By exposing the eDIB capsules to varying lysophosphatidylcholine (LPC) concentrations, we investigated the interaction of lysolipids with three-dimensional DIB networks. Micellar LPC concentrations triggered the bursting of encapsulated droplet networks, while at lower concentrations the droplet network endured structural changes, precisely affecting the membrane dimensions. This chemically-mediated manipulation of enclosed, 3D-orchestrated membrane mimics, facilitates the exploration of readily accessible compartmentalized artificial cellular machinery. Collectively, the droplet-based construct can pose as a chemically responsive soft material for studying membrane mechanics, and drug delivery, by controlling the cargo release from artificial cell chassis.

## Abstract (web summary)

Droplet interface bilayers can be used as a model of artificial membranes for synthetic biology and drug delivery applications, however, their accessibility using non-invasive techniques remains challenging. Here, the authors develop an in-situ bilayer manipulation of encapsulated droplet interface bilayers in hydrogel capsules, generated by high-order emulsification in monolithic 3D-printed microfluidic devices.

## Introduction

Droplet interface bilayers (DIBs) are bottom-up, cellular membrane-mimicking models used for the in-vitro study of membrane constituents and properties^1^. DIBs are formed when lipid monolayer-coated aqueous droplets come into contact, forming an artificial lipid bilayer membrane. In addition, DIBs can be formed when an aqueous droplet sits on top of a hydrogel substrate^2,3^, where this model has been used in single-molecule imaging for biophysical and biochemical studies^4,5^. The versatility of DIB models enables them to be tailored for different research applications, ranging from the study of transmembrane protein behavior^2^, to cell-free DNA expression^6^ and in-vitro tissue culture development^7^.

Sophisticated and functional artificial cellular networks can be constructed using DIBs as building blocks. Multisomes^8^, enclose DIB networks within an oil droplet, which can be suspended in air and water^9–11^. Various multisome demonstrations have been assembled using liquids only^8,11^, although, the encapsulation of DIBs and multisomes within soft hydrogels^12^, introduces soft material platforms towards the study of artificial membranes. Hydrogels are attractive because they are used for the immobilization of biological and non-biological matter, including living cells and synthetic cells, respectively^13,14^.

DIBs on hydrogel substrates acquire enhanced mechanical resistance leading to their prolonged stability and extended lifetime^12,15,16^. Gel-encapsulated droplet interface bilayer constructs (eDIBs)^17,18^ are a type of multisomes, which depict multi-compartmentalized artificial cell chassis and aim to impart cellular functionalities, such as polarization^19^. Furthermore, DIB systems are usually made by manual pipetting ^20^, which limits the production yield rate and structural complexity attained. Recently, multiphase microfluidic droplet-forming devices have been developed to effectively generate DIBs, multisomes and eDIBs, using stepwise emulsification methods^8,17,19^. Such droplet-based artificial membrane networks formed by robust and high-throughput microfluidic techniques have been used in molecular sensing^8^, cell mimicking^19^, and artificial cell membrane studies^17^.

The properties of simple and complex DIB systems are determined largely by the lipid and oil composition^21^, membrane chemistry ^22^, as well as the droplet arrangement^19,23^. Bilayer mechanics, forces and capacitance are characteristics directly influenced by the conditions of a DIB model^24–26^. Various studies have focused on the geometrical parameters of DIBs, e.g., contact angle and bilayer area, which are often manipulated, in order to modulate the behavior of the bilayer and transmembrane proteins^27^. An example includes mechanosensitive protein channels, whose activation relies on the tension across an asymmetric phospholipid bilayer^28–30^. This has been achieved using chemical means, such as the hydrolysis of lysolipids by phospholipase A_2_ or through physical actuation of the membranes^30,31^.

In addition to artificial cell studies, the manipulation of artificial phospholipid bilayers represents a cornerstone in the development of advanced biomimetic systems for biomedical applications, such as drug delivery and biosensing^32^. However, existing techniques often rely on invasive methods, which can compromise the integrity and functionality of the DIB structures. Invasive bilayer manipulation examples include the concentration minimization of protein pores and channels in DIBs, by directly dragging/pulling the droplets using electrodes or pipettes^27,28,33^. Others have induced liquid volume-assisted pressure changes within the DIB droplet-based compartments, therefore manipulating the droplet size and the bilayer area^34^. Alternatively, DIB manipulation has been achieved via electrowetting methods^24^, or through the incorporation of magnetic particles and exposure to magnetic fields^35^. Electrowetting manipulation of DIBs can be limited by electroporation and bilayer rupture^36^, while mechanical manipulation can be constrained by the contact and movement of invasive pipettes and electrodes, often causing failure of the DIBs.

Despite the widespread use of DIBs, the manipulation of these bilayers using non-invasive techniques remains an ongoing challenge. This study seeks to address this challenge by exploring a non-penetrating approach to control the behavior of artificial phospholipid bilayers, thus paving the way for more efficient and less disruptive biomedical and biotechnological applications. We propose a simple chemical approach to alter hydrogel-encapsulated DIB networks, to directly modulate the properties of artificial cells and enable the construct’s dynamic response to environmental changes. This concept is demonstrated by constructing eDIBs and observing their interaction with water-soluble lysophosphatidylcholine (LPC) for prolonged periods. These lysolipids are single-tailed phospholipids, which alter the surface tension of lipid monolayers and induce pressure changes along the phospholipid leaflet, as evidenced by artificial cell studies^37–39^. We find that at high concentrations (10-fold higher than the critical micellar concentration), LPC ruptures the artificial membranes and promotes rapid release of the enclosed aqueous content. At low, sub-micellar concentrations the droplet network endures physical changes, with significant alterations to the contact angle and bilayer area. Lysolipids were able to provide a facile and indirect contact approach for determining the fate of enclosed and interconnected DIBs in aqueous environments, making this system suitable for the active release of chemical species and non-invasive manipulation of artificial cellular membranes. Since in-situ controlled release from eDIB platforms can be established by utilizing the chemical sensitivity of functional elements (e.g., encapsulants or membranes), next-generation sensing and release technologies can be developed in macromolecular computing^40^, and can serve as biomimetic self-repairing materials ranging from biomedical implants to future constructional matrices^41^.

## Results and discussion

### High-order, gel-encapsulated DIBs using monolithic 3D-printed microfluidic devices

**Fig. 1:**
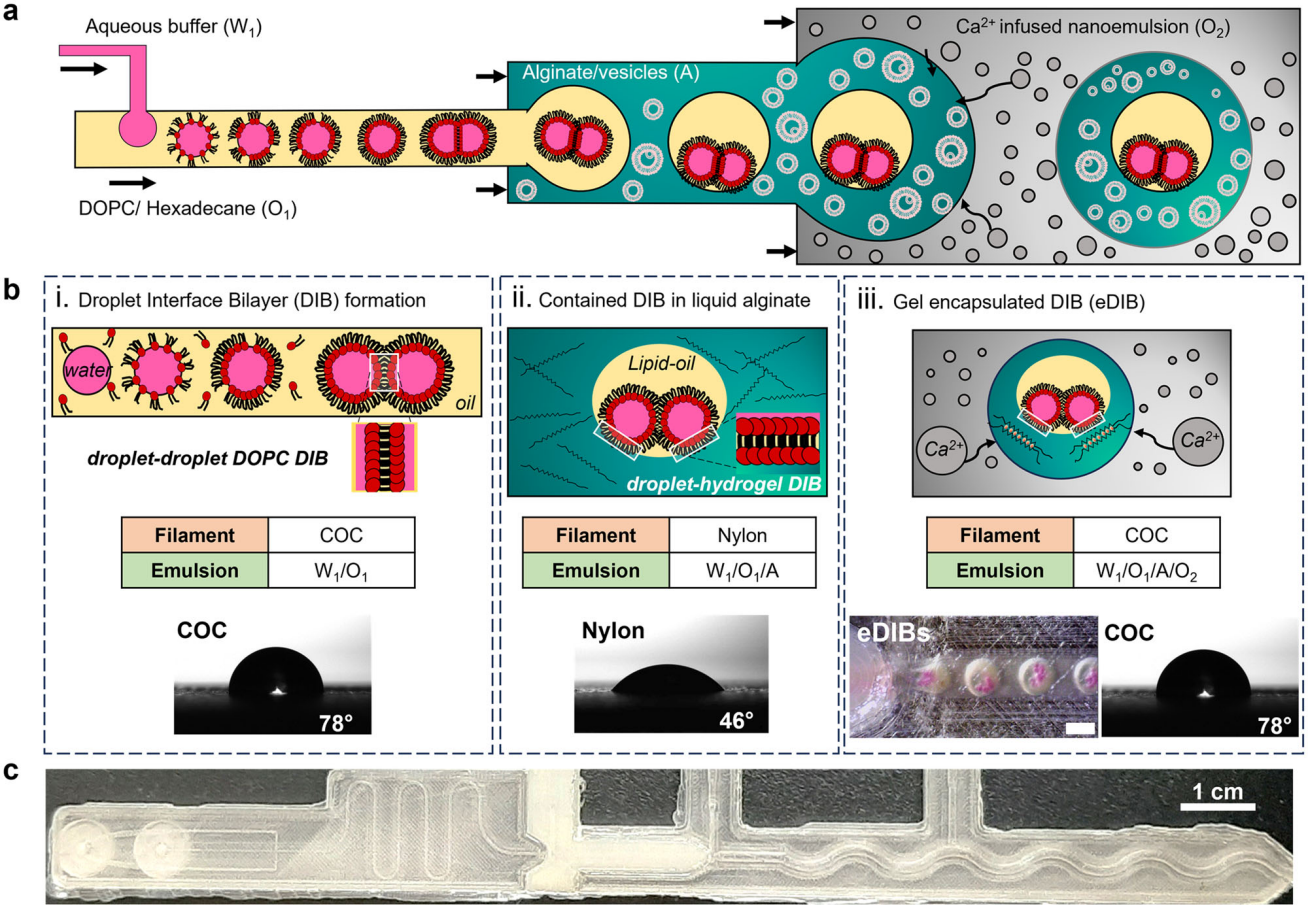
Monolithic 3D-printed microfluidic device generates triple emulsion capsules of encapsulated droplet interface bilayers (eDIBs). **a** Schematic of the triple emulsion microfluidic flow and production of eDIBs. The water phase (W_1_) is broken into droplets by the lipid-containing hexadecane oil (O_1_), which is then engulfed by a vesicle-containing alginate solution (A). The eDIBs are formed at the final 3rd junction and gelled downstream by the Ca^2+^-infused nanoemulsion (O_2_). **b** Schematics of the stepwise generation of eDIBs from (**a**) including filament type, contact angle, and emulsion order. i Water-in-oil (W_1_/O_1_) emulsion formed by the 1st hydrophobic (COC, 78°) droplet-forming junction. When the DOPC lipid monolayer-coated droplets come in contact, they form a DOPC droplet interface bilayer (DIB). ii A close look at a water-in-oil-in-alginate (W_1_/O_1_/A) emulsion formed at the 2nd hydrophilic (Nylon, 46°) droplet-forming junction. The DIB is contained by an alginate phase with DPPC vesicles (vesicles are not shown). Where an inner aqueous droplet contacts the alginate, another DIB is formed defined as a droplet-hydrogel DIB. iii The eDIB is formed at the 3rd droplet-forming junction (COC, 78°). The DIB contained by the alginate is engulfed by the Ca^2+^-infused nanoemulsion (W_1_/O_1_/A/O_2_), where the on-chip gelation starts (scale bar: 1 mm). **c** Picture of the 3D-printed COC-Nylon microfluidic device fabricated to generate the triple emulsion eDIBs capsules.

Three, in series, droplet-forming microfluidic junctions facilitated the formation of encapsulated DIBs in hydrogel capsules (Fig. 1a). For planar microfluidic devices, wettability is vital for successful and stable emulsion formation, which is usually achieved through channel surface modification, including plasma treatment and coatings^42^. Here, triple emulsion capsules were produced using a 3D-printed microfluidic device made from Nylon and Cyclic Olefin Copolymer (COC) without any surface treatment or other device post-processing. Nylon and COC polymers are known for their hydrophilic and hydrophobic surface property, respectively^43,44^. The surface water contact angle measurements of 3D-printed Nylon and COC substrates exhibited water contact angles of 46° and 78°, respectively (Fig. 1b, i–iii.). The print settings of each material (Supplementary Table 1) were kept consistent between all 3D-printed samples and microfluidic devices, as they can affect the final water contact angle of the substrate^45^. The Nylon and COC microfluidic components fused well together with no indication of leaking when the humidity was controlled while printing. It should be noted that Nylon fibers and films have been previously used in digital and paper microfluidics as superamphiphobic and anti-corrosive substrates^46–48^, however, Nylon is not widely used in droplet-microfluidics or 3D-printed microfluidics, possibly due to its hygroscopic properties^49^. Here, the 3D-printed Nylon microfluidic component offered a novel and facile method of producing high-order emulsions. In fact, Nylon filament is more suitable for dual-material 3D-printed microfluidic devices, since previously reported PVA devices were soluble in water, which limited the duration of the microfluidic experiments^19^. Earlier established eDIB models have been generated using glass capillary/3D-printed hybrid microfluidic devices^17^, or using double emulsion 3D-printed devices^19^.

The final microfluidic devices consisted of three droplet-forming junctions. The 1st and 3rd junctions were made of COC filament and the 2nd junction was made of Nylon filament. Initially, a water-in-oil (W_1_/O_1_) emulsion was formed at the 1st droplet-forming junction, which advantageously exploited the COC filament’s hydrophobic properties. In the oil (hexadecane), DOPC phospholipids were dissolved and resulted in the formation of a lipid monolayer around individual water droplets, which when in contact with each other, formed DIBs (Fig. 1b, i.). Subsequently, the W_1_/O_1_ inserted the 2nd droplet-forming junction made of Nylon filament (hydrophilic) and was broken by a continuous aqueous alginate phase. Therefore, multiple water droplets in lipid-containing oil (DIBs) were encapsulated in the liquid alginate, forming a water-in-oil-in-alginate (W_1_/O_1_/A) emulsion. At the site where an inner droplet comes in contact with the alginate, a droplet-hydrogel DIB is formed (Fig. 1b, ii.).

The lack of synthetic surfactants within the alginate resulted in the failure of the complex emulsion W_1_/O_1_/A. Instead of adding a surfactant into the alginate solution, we explored the addition of multilamellar DPPC vesicles, as surface tension-lowering agents^50,51^. This hindered the coalescence between miscible phases. Both DOPC and DPPC phospholipids have been used towards the construction of artificial cell membranes (e.g., liposomes), hence either DOPC or DPPC could be used in the alginate phase, however, only DPPC vesicles were studied here. Finally, the W_1_/O_1_/A was encapsulated by a divalent-infused nanoemulsion, for further emulsification (W_1_/O_1_/A/O_2_) and simultaneous on-chip gelation (Fig. 1b, iii.). The final constructs are referred to as eDIBs, as they are hydrogel-based constructs encapsulating DIBs and can be stored in an aqueous environment. To our knowledge, this is the first report of fabricating monolithic, 3D-printed microfluidic devices that can generate multi-compartment triple emulsions microgels, without performing any device post-fabrication treatment or processing (Fig. 1c).

**Fig. 2:**
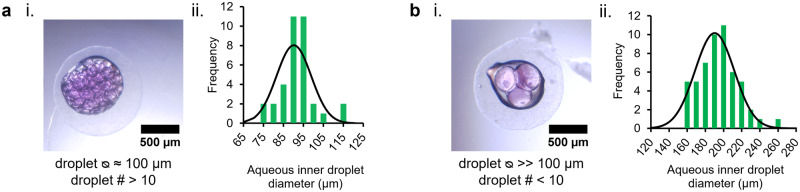
Gelled eDIB capsules with varying inner droplet diameter and number. **a** i eDIB capsule containing many droplets (# > 10) of small diameter (⍉ < 100 μm). ii Inner droplet diameter distribution plot of the eDIB in (i) (*n* = 35). **b** i eDIB capsule containing a small number of droplets (# < 10) of large diameter (⍉ > 100 μm). ii Inner droplet diameter distribution plot of the eDIB in (i). (*n* = 53). The gelled eDIBs shown were produced using the same microfluidic circuit and different flow rate combinations. Flow rates of (**a**, **b**) were 0.1 (W_1_): 0.5 (O_1_): 5 (A): 8 (O_2_) mL h^−1^ and 0.2 (W_1_): 0.2 (O_1_): 5 (A): 8 (O_2_) mL h^−1^, respectively.

Free-standing eDIB capsules were produced with varying numbers of inner droplets. By controlling the flow rates of the inner aqueous buffer and the DOPC-containing hexadecane phase we produced eDIBs with either an average diameter of 90 μm ± 1 μm (Fig. 2a) or 190 μm ± 3 μm (Fig. 2b). For reducing the diameter of the inner droplets, the aqueous phase flow rate of the inner droplets was decreased to 0.1 mL h^−1^, and the lipid-containing oil was increased to 0.5 mL h^−1^. eDIBs with smaller inner aqueous droplet diameters (⍉ < 100 μm) have been shown to be notably more robust after centrifugation (Supplementary Fig. 1).

It should be noted that often with 3D FFF printed micro-scale components, variabilities may be introduced on the microfluidic channel dimensions (Supplementary Table 2 and Supplementary Fig. 2), due to different environmental conditions and calibration inaccuracies. Because of these variabilities, eDIBs were formed at multiple phase flow rate combinations across experiments (Supplementary Table 3). For subsequent experiments the flow rates were manipulated accordingly, in order to enclose droplets with large diameter (⍉ > 100 μm) and a small droplet number (typically less than 10), which would permit good visualization of the droplet arrangement and DIBs. eDIBs that survive the initial 2–3 h of production can be stored for a month in an aqueous buffer with osmolarity that matches their internal droplets.

### Lysolipid-induced droplet release from eDIBs

Egg LPC is a water-soluble, cone-shaped, single-tailed phospholipid with a headgroup larger than the tail, which tends to form micellar lipid structures with positive curvature^52^. LPC has been used to alter the membrane pressure and activate mechanosensitive channels in DIB systems^30^, increase the permeability of cell membranes for drug uptake studies^53,54^, and facilitate protein pore insertion into bilayers^55^.

Here, the LPC lysolipid was introduced to the physiological aqueous environment surrounding the eDIBs capsules and diffused passively to the phospholipid DIB between the inner aqueous droplets and the hydrogel shell (droplet-hydrogel DIB). We consider that the LPC initially interacted with the outermost monolayer and later affected the inner monolayer, which will be explained in a later section.

**Fig. 3:**
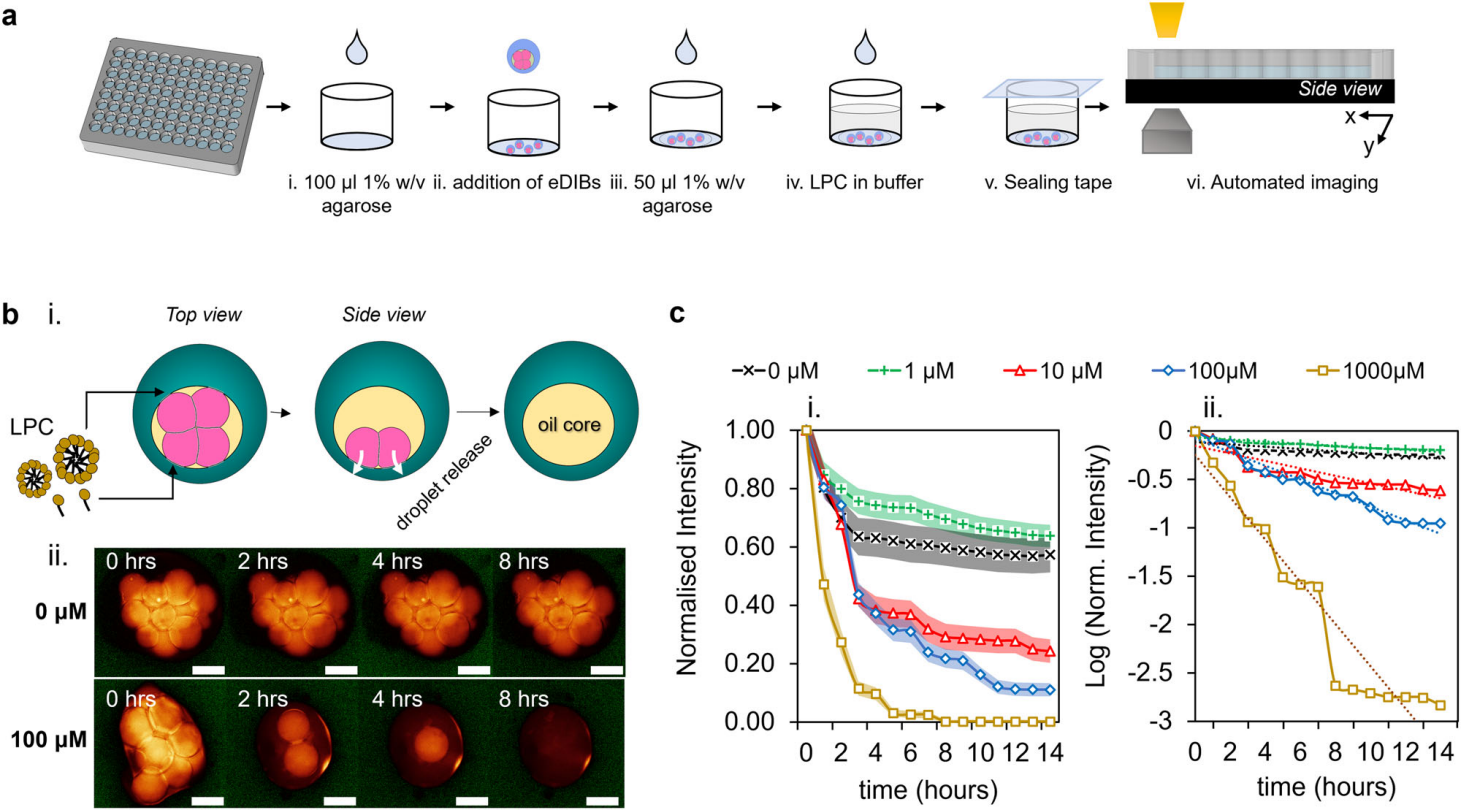
The effect of externally added LPC lysolipids on the release of inner aqueous droplets from eDIB capsules. **a** Stepwise schematic of the LPC treatment execution on eDIB capsules. First, a thin layer of 1% w/v agarose was added to the bottom of the well, followed by the addition of eDIB capsules and then another thin layer of agarose. This facilitated the immobilization of eDIBs at the bottom of the plate during the treatment and imaging with the EVOS automated platform. The temperature of the imaging platform was kept at 37 °C and the humidity was controlled by a well plate sealing tape. **b** i Top view and side view schematic of the eDIB capsules, showing the external addition of monomeric and micellar LPC. During the incubation of the eDIBs with concentrated LPC micelles, the lysolipids interact with DIBs formed between the hydrogel and inner aqueous droplets (droplet-hydrogel DIB) and subsequently, the droplets get released into the hydrogel. ii Time-lapse of the aqueous fluorescent (sulforhodamine B) inner droplets, showing the rapid release from eDIBs treated with micellar LPC concentrations (100 μM). Scale bar: 200 μm. **c** Fluorescent signal of the eDIB inner droplets incubated with different concentrations of LPC (fluorescent decrease assay). The intensity reduction for the untreated eDIB capsules (0 μM) is attributed to artefacts of the automated imaging platform and photobleaching. The sample population per concentration for the intensity analysis was as follows: *n* = 11 (0 μM), *n* = 15 (1 μM), *n* = 19 (10 μM), *n* = 17 (100 μM), *n* = 16 (1000 μM). i Normalized intensity versus time. The shaded regions for each line plot correspond to the standard error of mean (±SEM). ii The normalized intensity replotted in the logarithmic (log) scale over time. Besides this exponential decay, there are three consistent fluctuations at concentrations 10 μM, 100 μM, and 1000 μM showing a small delay with decreasing concentration. These fluctuations begin during a secondary process and finally level out.

Prior to imaging, the eDIBs were immobilized at the bottom of a 96-well plate using 1% w/v agarose, and this was followed by the addition of LPC in buffer at the final concentration of interest (Fig. 3a). The amphiphilic lysolipids diffused to the droplet-hydrogel DIB and at high concentrations (e.g., 100 μM) the inner droplets completely leaked into the surrounding medium, leaving an empty oil core (Fig. 3b). This was further analyzed in terms of the fluorescent signal drop over time, across a population of eDIB capsules exposed to various LPC concentrations (1–1000 μM). The droplet release profile for each concentration over a period of 14 h is shown in Fig. 3c, i. After ~3 h of incubation at 37 °C and constant humidity, the intensity of 0 μM and 1 μM LPC treated eDIBs stabilized with negligible reduction. This reduction of the fluorescent signal was attributed to possible photobleaching and out-of-focus imaging, caused by the moving platform. In addition, the inner droplets of eDIB capsules treated with 10 μM and 100 μM LPC were subject to major instabilities after ~2–3 h of the introduction of LPC. After the initial 3 h, the 10 μM LPC-treated eDIBs were able to maintain their stability for longer time periods compared to 100 μM LPC-treated eDIBs. The logarithm of the intensity revealed exponential decay over time with fluctuations at concentrations of 10 μΜ and higher, whilst it also uncovered the bursting events at concentrations of 1000 μM (Fig. 3c, ii).

The LPC composition used in this study was dominated by ~69% of 16:0 Lyso PC (information provided by the manufacturer), leading to the assumption that the critical micelle concentration (CMC) is close to that of 16:0 Lyso PC (CMC_LPC_). The CMC value is a variable of temperature, pH and salt^56,57^, and the exact CMC_LPC_ was not measured in this study. However, previous literature reported that the CMC_LPC_ value of 16:0 Lyso PC ranges between 4 μM and 8.3 μM, at temperatures spanning from 4 °C to 49 °C^58,59^. Therefore, only the 10 μM concentration introduced to the eDIBs in this study, was considered as a concentration closest to previously reported CMC_LPC_.

Either individual LPC lipid molecules, monomers (<CMC_LPC_), or micelles (>CMC_LPC_) were delivered to the droplet-hydrogel DIB and interacted with the first outer leaflet of the bilayer. This will alter the curvature of the membrane, leading to an asymmetric pressure distribution along the bilayer^60^. High micellar concentrations of LPC can lead to the rupture of phospholipid membranes, as a consequence of the translocation of crowded lysolipids to the second inner leaflet of the bilayer, or due to lysolipid-induced perturbations^54,61,62^. Similarly, here the droplets treated with equal to or greater than 100 μΜ LPC were subject to rapid droplet bursting, due to the failure of the droplet-hydrogel DIB membrane. In comparison to lower concentrations, this active release was attributed to the concentrated LPC micelles delivered to the targeted site (droplet-hydrogel DIB) and promptly induced membrane asymmetry. Supplementary fluorescence increase assays showed that 10 μM treated eDIBs underwent a major droplet-hydrogel DIB failure at a later timepoint (~7 h), compared to higher concentrations which caused instant membrane failure (Supplementary Fig. 3).

Lysolipid adsorption and insertion into the first monolayer is driven by diffusion at initially high rates and eventually slows down. In vesicles, LPC insertion can reach saturation of up to 10% within a few seconds and after this, LPC begins to translocate to the second monolayer^63^. The rate at which the saturation is reached is dependent on the initial concentration of LPC in the outer solution, whilst the rate at which exchange between the monolayers occurs, will depend on the concentration of LPC within the two monolayers. In complex artificial membrane models, including models such as the eDIB system, the LPC monomers may also flip between the bilayer midplane within hours of introducing sufficient concentrations of lysolipids^64^.

For lysolipids to diffuse and act on the droplet-hydrogel DIB, the monomers and micelles need to diffuse from the aqueous solution and then through the alginate shell. Lysolipids can interact and fuse with the DPPC lipid vesicles embedded in the hydrogel alginate shell, leading to the possible reduction of the lysolipid fraction delivered to the droplet-hydrogel DIB. An underestimated lysolipid concentration can influence the rate of impact on the eDIB constructs, which explains why the effects occur in the order of hours. Therefore, besides the ability to confine membrane networks, the hydrogel shell of the eDIB model works as a semi-permeable layer for controlling the diffusive transport of water-soluble reagents, e.g., LPC, and the associated interaction with the preformed DIBs. Furthermore, the micellar size highly depends upon the concentration, where 7-50 μM LPC forms micelles of 34 Å radius, whereas this micellar radius doubles at concentrations exceeding 50 μM^65^. Consequently, concentrations equal to or higher than 100 μM deliver large micelles, which contribute to the possible transient pore formation at the bilayer, thus the droplet-hydrogel DIB instantly fails and droplet release into the hydrogel occurs^37,66^. Taking into consideration the aforementioned, the concept of lysolipid-induced release from soft multi-compartmentalized eDIB capsules has potential applications in the delivery of highly organized chemical species, whose diffusion can be regulated by the internal structure and the physicochemical properties of the protective hydrogel shell.

### The effect of sub-micellar LPC concentrations on droplet displacement and arrangement

**Fig. 4:**
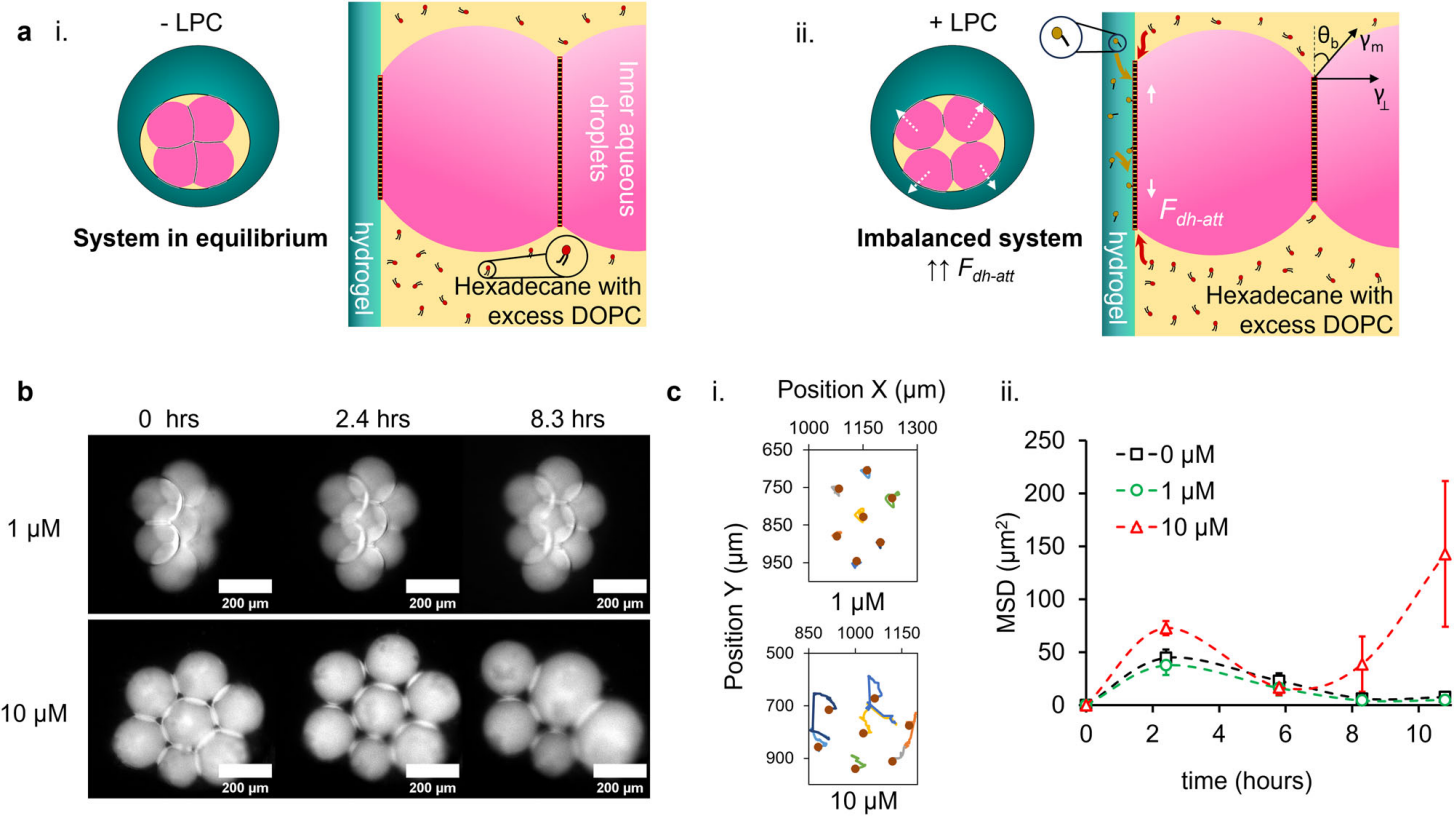
Inner droplet dynamics and re-arrangement under the influence of sub-micellar LPC lysolipid concentrations. **a** Schematic diagram of eDIBs and key bilayer interfaces before (−LPC) and after (+LPC) the addition of lysolipids. i The eDIB system and bilayer interfaces are at equilibrium, as attractive and repulsive forces balance each other. ii The introduced lysolipids take the eDIB out of equilibrium, as the LPC and DOPC contribute to the lateral expansion of the droplet-hydrogel DIB, by inserting from the external and internal side of the bilayer, respectively. Consequently, the attractive forces parallel to the droplet-hydrogel DIB$\,\left({\bar{F}}_{{dh}-{att}}\right)$ rise, due to the tensional changes at the outer bilayer. The contact angle (*θ*_b_) between the droplets is influenced by the increasing attractive forces at the droplet-hydrogel DIB. **b** Time-lapse of the inner aqueous droplets of eDIBs treated with 1 μΜ and 10 μΜ LPC, showing significant pulling and subsequent merging of droplets treated with 10 μM LPC. **c** Plots of the, (i) X and Y position of the inner droplets and, (ii) the mean square displacement (MSD) of 0 μM, 1 μM and 10 μM LPC treated eDIBs measured over 11 h, revealing that 1 μM treated droplets traveled similarly to the untreated construct, while there was significant travel by 10 μM treated droplets. The dots in (i). show the location of the individual droplets at *t* = 0. Error bars in (ii). correspond to the standard error of the mean (±SEM).

Lipid monolayer-coated aqueous droplets in the form of water-in-oil emulsion are governed by the interfacial tension. Bilayer and DIB formation is facilitated by Van-der-Waals forces, as the adhesive lipid monolayer-coated droplets come in contact^67,68^. During DIB formation there are temporary fluctuations of the disjoining pressure at the artificial membrane^68^, but the attractive and repulsive forces work towards the equilibrium of the system^69–71^. The eDIBs presented in this study reach equilibrium in a similar manner and become unstable once lysolipids are introduced. Significant disturbances begin when the lysolipids are externally introduced to the eDIBs, as illustrated in Fig. 4a, due to the asymmetry introduced in the membrane. The equilibrium is destabilized, as the introduced lysolipids begin to feed into the existing outermost phospholipid bilayer of the eDIB^72^. The organization of the LPC lysolipids within the first encountered lipid monolayer of the droplet-hydrogel bilayer results in the reduction of the monolayer tension on one side of the bilayer^73^. The reduction of the outer monolayer tension causes the overall bilayer to expand laterally. The excess of DOPC lipids in the encapsulated oil core phase is also considered to contribute to the membrane expansion from the inner side of the bilayer (Fig. 4a, ii). Therefore, both leaflets endure tensional changes, which causes the adhesive forces directed along the interface of the droplet-hydrogel bilayer to shift and the whole bilayer to expand along the interface.

In the presence of lysolipids, we classify the dominating forces acting parallel to phospholipid bilayers of the eDIB model. These primarily consist of the attractive forces at the droplet-hydrogel DIB$\,\left({\bar{F}}_{{dh}-{att}}\right)$. As the droplet-hydrogel bilayers laterally expand in the presence of LPC, the ${\bar{F}}_{{dh}-{att}}$ dominate over any other attractive forces (Fig. 4a, ii). This is demonstrated by the pulling of the droplets towards the hydrogel shell, evidencing changes in the monolayer and bilayer composition at the droplet-hydrogel interface. Here, we describe the pulling effect as the retraction of the droplets away from the center of the middle oil core. The membrane expansion, accompanied by the fact that the inner droplets are of limited volume and encased in a confined space, leads to their attraction towards the outer droplet-hydrogel interface. At this stage, the inner droplets are forced to follow the direction of the area of bilayer expansion as the monolayer tension decreases, resulting in the reduction of the droplet-droplet bilayer area interface. These changes will promote the merging of the inner droplets between each other or with the outer hydrogel. If LPC monomers translocate from the outermost monolayer to the inner monolayer at a rate that the membrane system can withstand, equilibrium might be reached again, as established by 1 μM treated eDIBs.

The above molecular dynamic LPC-induced changes promote the rearrangement and displacement of the inner droplets and DIBs. The rate and the degree of destabilization effects depended on the concentration of LPC introduced. Droplet pulling was more explicit in eDIBs treated with 10 μM LPC, as shown in Fig. 4b. eDIBs treated with 1 μM LPC were overall less disturbed with mean square displacement similar to the control (0 μM), while the displacement of the droplets exposed to 10 μΜ LPC was more apparent (Fig. 4c). After ~8 h of incubation with the lysolipids, the pulling effect led to droplet merging in eDIBs treated with 10 μM LPC. In fact, during the study period and at this concentration of LPC, it was observed that the inner droplets would initially merge between them, and not with the hydrogel shell. This was due to the enhanced stability of DIBs formed on hydrogel semi-flat substrates^74^, compared to droplet-droplet DIBs. Once the first merging occurred, a cascade of merging continued where small droplets merged with larger droplets (the product of merging), due to the higher Laplace pressure inside smaller droplets^74^. The delayed droplet shifting and displacement in the presence of 10 μM LPC (Fig. 4c, ii) were attributed to the slower build-up of lysolipid concentration at the droplet-hydrogel DIBs^75^.

### The effect of sub-micellar LPC concentrations on DIB bilayer area and contact angle

High bilayer tension and strong adhesion forces are associated with increased bilayer area and contact angles in DIB systems^23,76^. In this study, LPC promoted compositional changes to the monolayer and bilayer at the droplet-hydrogel DIB, giving rise to the movement of the inner droplets towards the hydrogel and changes in the membrane dimensions of the internal bilayers. The bilayer area and contact angle were not captured at the droplet-hydrogel DIB, due to imaging limitations, and were only measured between the aqueous droplet-droplet DIBs.

**Fig. 5:**
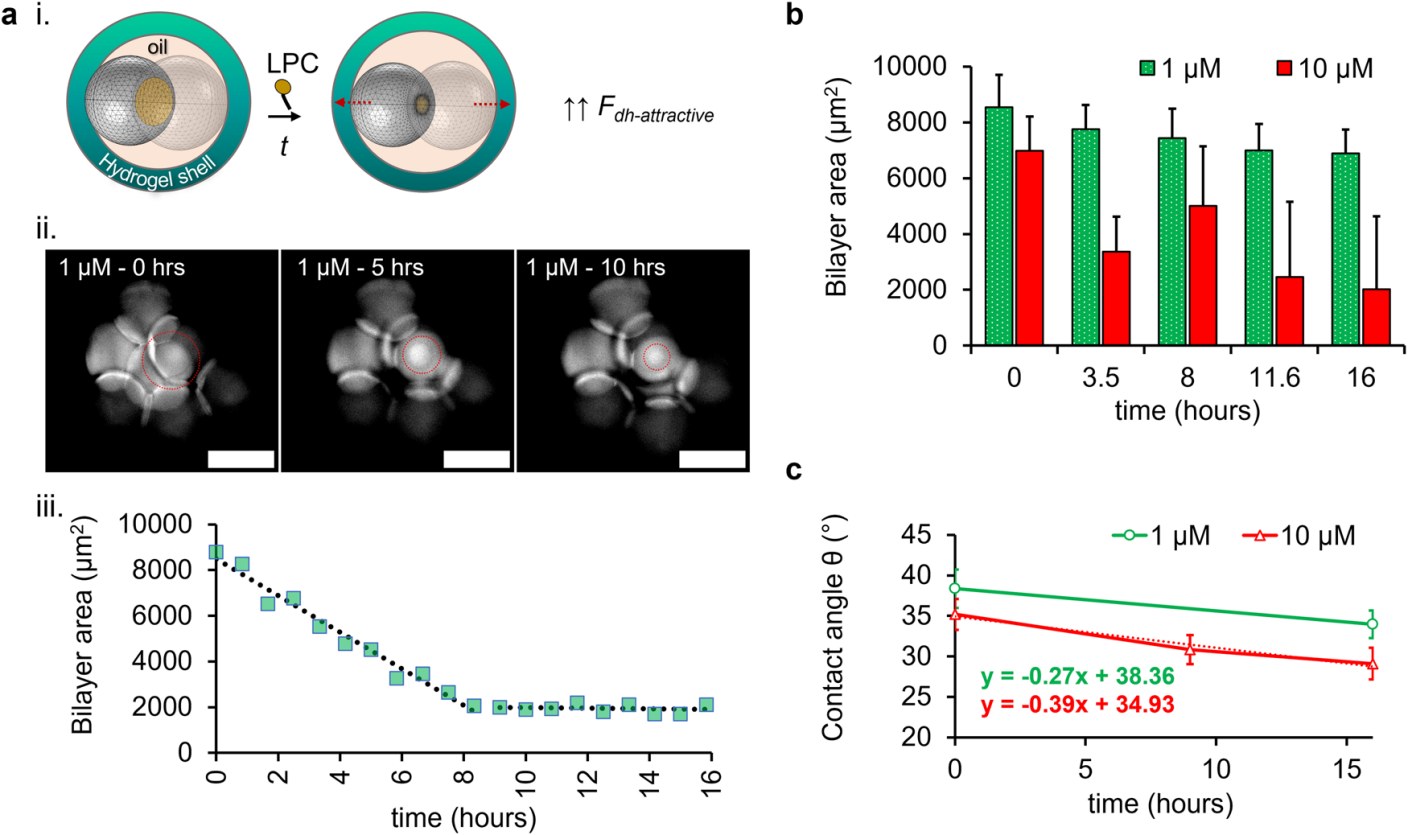
LPC lysolipid impact on the bilayer area and contact angle of eDIBs. **a** i A schematic of an eDIB capsule with two inner droplets and a formed DIB (yellow circular droplet contact area), before and after the addition of LPC. The DIB area is reduced during incubation with LPC, as the adhesive forces of the droplet-hydrogel bilayer begin to dominate (↑↑ F_dh-attractive_), due to the compositional changes in the outer monolayer and bilayer. ii Time-lapse of fluorescent droplets encapsulated within an eDIB capsule treated with 1 μM LPC, showing the reduction of the bilayer area as indicated by the red dotted circle. To reveal the bilayer between the contacting droplets, the brightness and contrasts of the image were adjusted. Scale bar: 200 μm. iii The measured circular bilayer area from (ii) is plotted over time as a scatter plot, whilst the dotted curve shows the linear decrease in the first 8 h after 1 μM LPC addition; this is followed by a transition to a constant bilayer area (equilibrium reached) until the end of the study. **b** Average DIB bilayer area over time across a population of eDIBs treated with 1 μM (*n* = 11, *N* = 4) and 10 μM (*n* = 12, *N* = 5) LPC. The DIB bilayer area of 10 μΜ treated constructs displays a drop at 3.5 h and then an increase at ~8 h, which indicates first the pulling of the droplets and subsequent merging, respectively. After that, the bilayer area follows a reduction and begins to equilibrate. A minimal and subtle decrease was observed in the bilayer area throughout the study in 1 μM treated eDIBs. The number of measured vertical bilayers for 10 μM treated DIBs was initially *n* = 12 (*N* = 5), and this dropped to *n* = 4 (*N* = 5) by the final timepoint, due to droplet merging. **c** Line graph of the average DIB contact angle as a function of time for 1 μΜ (*n* = 55, *N* = 6) and 10 μΜ (*n* = 47, *N* = 9) treated eDIB capsules. An additional timepoint at ~9 h was plotted, which corresponds to the initial merging of droplets treated with 10 μΜ (best fit for 10 μM treated eDIBs shown by the dotted line). The line plots are accompanied by linear equations, which reveal the initial average DIB contact angle (38° for 1 μM and 35° for 10 μM). The population number of the measured contact angles for 10 μM was *n* = 47 (*N* = 9), and this dropped to *n* = 22 (*N* = 9) by the final timepoint, due to droplet merging. The number of eDIBs is noted by *N*, whilst the sample population of the measurable characteristic (bilayer area or contact angle) is noted by *n*.

The three-dimensional micro-architecture of the eDIB capsules benefited the measurements of circular bilayer areas, which reflect the shape of the droplets, throughout incubation as shown in Fig. 5a, i–ii. This allowed the quantification of circular bilayer areas of DIBs between adjacent droplets, which helps assess the bilayer stability and behavior^16^. The bilayer area of 1 μM treated eDIBs shows delayed effects induced by LPC and subsequent return to equilibrium, as evident by the bilayer area plateau (Fig. 5a, iii). Moreover, the bilayer area of vertical droplet-droplet DIBs inside eDIB capsules was calculated on the assumption that the droplets on either side of the bilayer were of equal diameter (Supplementary Fig. 4*)*. Figure 5b shows the average bilayer area of 1 μM and 10 μM treated DIBs throughout the incubation period. Whilst negligible bilayer area reduction was observed with 1 μM LPC, the 10 μM LPC caused a significant reduction within the initial 3.5 h, followed by droplet merging (bilayer area increase) and then once again, bilayer area reduction.

The contact angle of DIBs typically depends on the droplet diameter, lipids and oil composition, as they can affect the surface tension and consequently the droplet-droplet adhesion^21^. The number of droplets enclosed within a volume forming DIBs can also affect the droplet-droplet contact angle^23^. In most DIB models, the contact angle of DIBs is manipulated prior to the DIB formation by varying the lipid and oil composition (Supplementary Fig. 5*)*. In this study, the contact angle among the inner compartments can be manipulated post-fabrication through the incubation of the eDIBs with sub-micellar LPC concentrations. This is displayed in Fig. 5c., where the mean contact angle between the eDIB inner droplets was measured before the LPC started to affect the droplet-droplet DIBs to a measurable extent, and at the end of the incubation. In addition to the endpoint contact angle measurements, the contact angle was measured at ~9 h for eDIBs only treated with 10 μM LPC, representing an average timepoint after the first droplet coalescence.

Bilayer peeling between two droplets of contact angle$\,{\theta }_{b}$ and monolayer surface tension $({\gamma }_{m})$ was previously attributed to the exceeding of the critical adhesive bilayer force per unit length, by a quantity $\left({\gamma }_{\perp }\right)$ which drives the droplet-droplet DIB separation, ${\gamma }_{\perp }=\,{\gamma }_{m}\sin {\theta }_{b}$^25^. Huang et al. ^26^ studied this separation quantity by mechanically pulling one droplet of the DIB, while our study shows peeling without invasively interfering with the system^25^. The lysolipids alter the composition of the outer monolayer of eDIBs and bilayer following translocation, pulling the internal droplets towards the hydrogel, hence changing the ${\theta }_{b}$ and membrane dimensions between adjacent internal droplets. This gives rise to the separation quantity ${\gamma }_{\perp }$, opposite the movement of the droplet (Fig. 4a, ii). Here, the peeling at the droplet-droplet DIB is dominated by the tensional changes at the expanding outer monolayer and droplet-hydrogel bilayer. Dimensional changes at the droplet-droplet DIBs were conveniently measured as the droplets were constrained in an oil core. Therefore, the eDIB model presents a durable platform to study the influence of contact angle and membrane dimension versus the aspect ratio of the encapsulated droplets.

Overall, we demonstrated the generation of encapsulated droplet interface bilayer membranes into self-supported hydrogel capsules (eDIBs) using 3D-printed COC/Nylon microfluidic devices. These materials facilitated the stepwise emulsification of the triple emulsion eDIB constructs. This was also benefited by utilizing lipid vesicles in the hydrogel precursor, as interfacial tension-altering particles, hindering the mixing between miscible phases. Alginate eDIB capsules were crosslinked using an on-chip calcium-infused nanoemulsion and this contributed to maintaining the internal artificial membrane network. An active content release approach from eDIBs was established by introducing LPC at micellar lysolipid concentrations. On the other hand, sub-micellar concentrations induced more refined effects, including 3D reorganization and changes in the bilayer area and contact angle, which implies bilayer remodeling and thus tensional changes of the outermost monolayer and bilayer.

Advantages for employing microfluidics in DIB model construction include the high production yield, and control over the size and structural order, whilst various features can be introduced, such as phospholipid bilayer asymmetry. The incorporation of phospholipid DPPC vesicles within the alginate phase can contribute towards the formation of asymmetric DOPC/DPPC bilayers, following partial lipid-in and lipid-out DIB formation. Although, in this study, we considered a lipid-out symmetric DOPC bilayer constructed at the bilayer interface between droplets, and between any droplet and the hydrogel.

A previous study on hydrogel eDIBs demonstrated the synergy between pore-forming peptides, but no control over the organization or DIB adhesion was reported^77^. Furthermore, when cholesterol molecules insert between the phospholipids of a bilayer, they create a condensed monolayer with restricted motion between the acyl chains of the phospholipids^71^. Similar to cholesterol molecules, lysolipids at non-pore-forming concentrations insert between phospholipid molecules and alter the surface tension of the phospholipid monolayer (for LPC, tension will be higher between polar headgroups), and the energy of adhesion of the bilayer.

For the duration of the lysolipid LPC treatment, we hypothesized that the LPC molecules introduced to the eDIB system were unable to encounter the droplet-droplet DIB, directly. Therefore, the lysolipids only affected the droplet-hydrogel interface through the translocation of lysolipids across the midplane of the outermost bilayer. The monolayer and bilayer phospholipid compositional changes at the droplet-hydrogel interface led to strong adhesion forces to pull the droplets and attenuate the droplet-droplet DIB area. These findings present an approach for in-situ and automated organization, as well as the manipulation of the bilayer area and contact angle of encapsulated droplet-droplet DIBs. It should be noted that the duration of phospholipid bilayer exposure to lysolipids can enhance the lipid molecular transfer to the opposite leaflet^63^ and hence, the degree of impact.

Research in artificial cells and protein reconstitutions would benefit from the non-invasive modulation of artificial cellular membranes. Simply by introducing lysolipids, the spatial organization and physicochemical characteristics of membranes can be modulated, offering a new approach to manipulating artificial cellular materials with multiple compartments and encapsulants. These findings pave the way for non-invasive transmembrane protein density control in three dimensions, as well as the modulation of chemically mediated communication pathways among artificial cell chassis and surrounding aqueous environments. Droplet microfluidic technology provides a versatile tool for engineering increasingly sophisticated droplet structures, which can serve as artificial membrane models to study biomolecular interactions and precision engineering of interconnected bioinspired membranes. Engineering and exploring novel biomimetic platforms and their integration with synthetic biology can advance the development of responsive soft matter with potential applications in controlled drug release systems.

## Materials and methods

### Materials

COC was purchased from Creamelt (Grade 8007, TOPAS), and transparent Nylon was purchased from Ultimaker. Sulforhodamine B and calcein were purchased from Thermofisher, UK. The calcein and sulforhodamine were dissolved in 0.05 M HEPES, 0.15 M KCl in deionised water (buffer) or Phosphate Buffered Saline, PBS (pH 7.4, 1X, Gibco, UK). Alginic acid sodium salt from brown algae, hexadecane, silicone oil AR20, mineral oil, calcium chloride, HEPES, potassium chloride, 1,2-di-oleoyl-sn-glycero-3-phosphocholine (DOPC), 1,2-dipalmitoyl-sn-glycero-3-phosphocholine (DPPC), Egg LPC, chloroform and SPAN 80 were purchased from Merck. The average fatty acids in the egg LPC mixture according to the manufacturer was 69% 16:0, 24.6% 18:0, 3.4% 18:1, 1.4% 16:1, 0.3% 14:0, 0.3% 18:2, and 1% unknown.

### 3D-printed microfluidic device fabrication and operation

The microfluidic device was designed using COMSOL Multiphysics (version 5.6) and fabricated using the Ultimaker S5 Pro Bundle with Cyclic Olefin Copolymer (Creamelt) and Nylon (Ultimaker). The device was sliced using the CURA software with the assigned print settings summarized in Supplementary Methods. All devices after printing were stored with silica gel sachets. Each liquid phase was delivered to the microfluidic device using SGE gas-tight glass syringes loaded onto positive displacement syringe pumps (KD Scientific). The SGE syringes were connected directly to the 3D-printed microfluidic inlets using PTFE tubing (O.D. ⍉ = 1.58 mm, I.D. ⍉ = 0.80 mm). Further details regarding the microfluidic device, channel dimensions, and flow operation can be found in Supplementary Methods.

### Production of water-in-oil-in-alginate-in-oil eDIB capsules (W_1_/O_1_/A/O_2_)

All reagents were purchased from Merck, unless otherwise stated. The inner water phase (W_2_) consisted of a buffer solution of 0.05 M HEPES, 0.15 M potassium chloride, 200 μM of sulforhodamine B (SulfB) or 70 mM calcein. The middle oil phase (O_1_) consisted of 12.5 mg mL^−1^ 1,2-di-oleoyl-sn-glycero-3-phosphocholine (DOPC) in hexadecane. DOPC was first dispersed in hexadecane following the thin film lipid hydration method. Briefly, the DOPC powder was dissolved in chloroform and evaporated using a gentle nitrogen stream until a thin film of lipids was formed. The DOPC film was subject to a vacuum for at least 30 min to evaporate any residual chloroform and then released under nitrogen gas. The shell phase (A) consisted of 1% w/v alginate and 0.5 mg mL^−1^ 1,2-dipalmitoyl-sn-glycero-3-phosphocholine (DPPC) vesicles in the buffer. The DPPC vesicle solution was prepared using the thin film lipid hydration method, following vacuum overnight. The DPPC film was dispersed in the buffer solution, vortexed for 30 s and sonicated in a water bath at 55 °C for 15 min. The eDIB capsules’ oil carrier phase (O_2_) consisted of a Ca^2+^ - infused mineral oil emulsion, which facilitated the gelation of the alginate shell. This carrier phase was prepared by mixing an aqueous solution of 1 g mL^−1^ CaCl_2_ and mineral oil at 1:9 volume ratio, with 1.2% SPAN 80 surfactant. The mixture was stirred for at least 10 min using a magnetic stirrer and plate, creating a Ca^2+^-infused nanoemulsion. During experiments, the outlet orifice was slightly submerged in 0.2 M CaCl_2_.

The microfluidic setup and execution here, aimed at the formation of ~1 mm diameter eDIBs, with large water droplet compartments (>100 μm) segregated by artificial lipid membranes (i.e., DIBs).

### LPC treatment of eDIBs

eDIB capsules were immobilized with 1% w/v low-temperature melting agarose in wells of a 96-well plate. LPC in buffer was prepared and used appropriately, in order for each well to have a final LPC concentration of 1, 10, 100, and 1000 μM. The droplet release was evaluated by monitoring the decrease in the fluorescence of sulfB (200 μM) from the droplets of individual eDIBs or the fluorescence increase in the wells with eDIBs encapsulating quenched calcein (70 mM). Details related to the LPC fluorescence increase assay can be found in the Supplementary Methods.

### Optical and fluorescence microscopy of eDIBs

eDIBs during on-chip emulsification were imaged using Dino-Lite edge USB microscope. eDIBs post-production and during the LPC treatment were imaged using the EVOS M7000 Imaging System (TxRed and GFP LED cubes). Imaging associated with the lysolipid treatment was carried out at 37 °C, where the well plate containing the eDIB capsules was sealed with tape to prevent evaporation.

### Bilayer area and DIB contact angle measurements

The bilayer area was measured in three different ways depending on the bilayer orientation and sphericity of the droplets forming the DIB. See the Supplementary Methods section for bilayer area calculations. Due to the ability of the inner droplets to maintain their three-dimensionality, the contact angle was simply calculated by measuring the angle between two adjacent inner aqueous droplets using the angle drawing tools on ImageJ. Before measuring the angle, the contrast of the image was adjusted accordingly, in order to remove any noise around the region of interest. The bilayer area and contact angle of eDIBs produced using 4 mg mL^−1^ DOPC in 10% silicone oil were also measured as reference and comparison to conventionally produced eDIBs (12.5 mg mL^−1^ DOPC, 100% hexadecane).

### Fluorescence and image analysis

The droplet release in the fluorescence decrease assay was evaluated by monitoring the fluorescence decrease from the aqueous sulfB droplets of individual eDIBs (measured the area of the fluorescent DIBs inside the whole construct.). The droplet release in the fluorescence increase assay was evaluated by monitoring the fluorescence increase of the wells with the eDIBs carrying droplets of quenched calcein (70 mM). Image handling and fluorescence analysis were carried out using ImageJ software. The integrated fluorescent intensity was measured at the time point of interest, with the ROI minimized to the area of the fluorescent droplets. The intensity plots show the intensity normalized to the intensity extracted from the control (0 μM LPC) fluorescent droplets in eDIBs. The position and displacement of the droplets were recorded using manual tracking tools within ImageJ. The eDIB samples were monitored for over 10 h and the position of the droplets was recorded every 5 min.

### Supplementary information

Supplementary Information

Description of Additional Supplementary Files

Supplementary Data

## Data Availability

All data supporting the findings of this study are available within this article, the Supplementary Information, and the Supplementary Data.
