# Supplementary material for: Manipulation of encapsulated artificial phospholipid membranes using sub-micellar lysolipid concentrations
Source: Commun Chem. 2024 Jun 1;7:120. doi: 10.1038/s42004-024-01209-z (PMC11144220; doi:10.1038/s42004-024-01209-z)
Supplement: Supplementary file 2 — Description of Additional Supplementary Files [file 42004_2024_1209_MOESM2_ESM.pdf]

Description of Additional Supplementary Files

File name- Supplementary Data

File description- Supplementary Data for graphs in Figure 2a-b, 3c, 4c, 5a-c.
